# Imaging study of coccygeal morphology in adolescent idiopathic scoliosis

**DOI:** 10.1038/s41598-024-51276-4

**Published:** 2024-01-06

**Authors:** Haoyang Zhang, Yingsen Pan, Chenhao Ju, Yi Shen, Xiaoming Li, Xin Ye, Binghua Fan, Shuailin Li, Yongliang Jiang, Benshun Yao, Xiaoming Ying

**Affiliations:** 1 The 3rd Clinical Medical College of Zhejiang, Chinese Medical University, Hangzhou, China; 2 Tuina Department, The 3rd Affiliated Hospital of Zhejiang Chinese Medical University, Hangzhou, China; 3 Department of Acupuncture and Massage, Hangzhou Binjiang Hospital of Traditional Chinese Medicine, Hangzhou, China

**Keywords:** Paediatric research, Bone

## Abstract

The correlation between scoliosis and sagittal curvature of the cervical, thoracic, and lumbar spine have already been reported in previous studies. However, as a part of the spine, the change in coccygeal morphology in AIS patients has not yet been studied. In this study, a retrospective analysis was performed on 400 patients who were divided into a non-scoliotic group (206 patients) and an AIS group (194 patients). The Postacchini coccygeal radiological classification that was modified by Nathan was used to observe and compare the sagittal coccygeal morphology between the two groups. The results showed that the non-scoliotic group had the highest percentage (52.4%) of patients with type I and the lowest (3.4%) proportion of patients with type V; moreover, the AIS group had the highest percentage (69.1%) of patients with type I and the lowest (1.5%) proportion of patients with type V. The coccygeal morphology was significantly different between the non-scoliotic group and the AIS group (*P* = 0.001). No significant differences in coccygeal morphology were found between the males and females in the two groups (mild and moderate scoliosis and different segmental scoliosis). In addition, a significant correlation between coccygeal morphology and scoliosis (*P* = 0.035) was found. In conclusion, coccygeal morphology significantly differs between AIS patients and non-scoliotic adolescents. There was a smaller proportion of patients with a type I coccyx and a larger proportion of patients with a type II or type III coccyx in the AIS group than in the non-scoliotic group. In other words, the presence of a more pronounced coccygeal curve in AIS patients may be caused by an incorrect sitting position and an imbalance in the contraction of the pelvic muscles. It should be further studied whether correcting the sitting position and muscular imbalances could change coccygeal morphology and subsequently affect the development of AIS.

## Introduction

Adolescent idiopathic scoliosis (AIS) is a prevalent condition that accounts for more than 85% of all scoliosis cases and affects 2 to 3% of adolescents^1,2^. Recent survey data showed that AIS was the third most common disease among adolescent children in China, following obesity and myopia. This condition has a significant impact on the physical and mental health of young people^3^. Scoliosis is classified as a “three-dimensional rotational deformity of the spine and trunk”^4^. Despite a lack of understanding of the pathogenesis of AIS, it is widely recognized that, in the sagittal plane, the physiological curvature of the spine is a contributing factor to spinal function and scoliosis progression^5–7^. Hilibrand et al.^8^ reported that AIS patients had notably smaller cervical lordosis angles than typical adolescents. Similarly, Hiyama et al.^9^ reported that approximately 59.5% of AIS patients exhibited deviations in cervical curvature, ranging from decreased lordosis to reversed kyphosis. Furthermore, the angles representative of thoracic kyphosis (TK) and lumbar lordosis (LL) were well-established and frequently applied^10–12^. Moreover, extensive research has been conducted on the sagittal parameters of the spine‒pelvis relationship in AIS patients^13,14^. A study showed that pelvic parameters were important indices for assessing pelvic morphology, and the pelvic-spinal system was a crucial factor affecting scoliosis^15^. The coccyx, which is the furthest distal component of the spine, consists of multiple segments^16,17^. As an important component of the pelvic structure, the coccyx may have an impact on the progression of spinal scoliosis. However, changes in coccygeal morphology in AIS patients have not yet been studied. Therefore, the authors in the present study hypothesize that coccygeal morphology would be different in AIS patients. The purpose of this study is to observe the coccygeal morphology in AIS patients and the differences among different types of scoliosis.

## Results

### Comparison of coccygeal morphology between the two groups

There were 206 patients in the non-scoliotic group and 194 patients in the AIS group. The percentages of type I-V patients in the non-scoliotic group were 52.4%, 17.5%, 14.6%, 12.1%, and 3.4%, respectively. The percentages of type I-V patients in the AIS group were 33.5%, 28.9%, 22.1%, 13.9%, and 1.5%, respectively. There was a statistically significant difference in coccygeal morphology between the two groups (*P* = 0.001; Table 1).

**Table 1:** Comparison of coccygeal morphology between two groups (cases/%).

	Type I	Type II	Type III	Type IV	Type V	χ2	*P*
Normal group	108/52.4	36/17.5	30/14.6	25/12.1	7/3.4	18.684	0.001
AIS group	65/33.5	56/28.9	43/22.2	27/13.9	3/1.54		

### Comparison of coccygeal morphology between mild and moderate AIS patients

There were 139 patients with mild AIS and 55 patients with moderate AIS in the present study. The percentages of type I-V patients in the mild AIS group were 36.0%, 26.6%, 22.3%, 13.7%, and 1.4%, respectively, and the percentages of type I-V patients in the moderate AIS group were 27.3%, 34.5%, 21.8%, 14.5%, and 1.8%, respectively. There was no significant difference in coccygeal morphology between the mild and moderate AIS groups (*P* = 0.771; Table 2).

**Table 2:** Comparison of coccygeal morphology between mild and moderate AIS (cases/%).

	Type I	Type II	Type III	Type IV	Type V	χ2	*P*
Mild	50/36.0	37/26.6	31/22.3	19/13.7	2/1.4	1.810	0.771
Moderate	15/27.3	19/34.5	12/21.8	8/14.5	1/1.8		

### Comparison of coccygeal morphology among different types of segmental scoliosis

The coccygeal morphology was compared among patients with different types of segmental scoliosis. There were 95 patients with thoracic scoliosis, 32 patients with thoracolumbar scoliosis, and 67 patients with lumbar scoliosis in the study. The percentages of type I-V patients in the thoracic AIS group were 31.6%, 28.4%, 26.3%, 11.6%, and 2.1%, respectively; the percentages of type I-V patients in the thoracolumbar AIS group were 46.9, 28.1%, 15.6%, 9.4%, and 0%; and the percentages of type I-V patients in the lumbar AIS group were 29.9%, 29.9%, 19.4%, 19.4%, and 1.4%, respectively. The difference in coccygeal morphology among the different segmental scoliosis subtypes was not significant (*P* = 0.571; Table 3).

**Table 3:** Comparison of coccygeal morphology among different segmental scoliosis (cases/%).

	Type I	Type II	Type III	Type IV	Type V	χ2	*P*
Thoracic	30/31.6	27/28.4	25/26.3	11/11.6	2/2.1	6.685	0.571
Thoracolumbar	15/46.9	9/28.1	5/15.6	3/9.4	0/0		
Lumbar	20/29.9	20/29.9	13/19.4	13/19.4	1/1.4		

### Comparison of coccygeal morphology between males and females in the two groups

The non-scoliotic group comprised 109 males and 97 females. The proportions of type I to type V patients who were male were 56.9%, 17.4%, 15.6%, 6.4% and 3.7%, respectively, and who were female were 47.4%, 17.5%, 13.4%, 18.6% and 3.1%, respectively. There were no significant differences between males and females in the non-scoliotic group (*P* = 0.120; Table 4). There were 147 female patients and 47 male patients in the AIS group. The proportions of type I-V male patients were 31.9%, 29.8%, 25.5%, 10.6%, and 2.1%, respectively; the proportions of type I-V female patients were 34.0%, 28.6%, 21.1%, 15.0%, and 1.4%, respectively. The difference in coccygeal morphology between females and males in the AIS group was not statistically significant (*P* = 0.910; Table 5).

**Table 4:** Comparison of coccygeal morphology between male and female in normal group (cases/%).

	Type I	Type II	Type III	Type IV	Type V	χ2	*P*
Male	62/56.9	19/17.4	17/15.6	7/6.4	4/3.7	7.723	0.120
Female	46/47.4	17/17.5	13/13.4	18/18.6	3/3.1

**Table 5:** Comparison of coccygeal morphology between male and female in AIS group (cases/%).

	Type I	Type II	Type III	Type IV	Type V	χ2	*P*
Male	15/31.9	14/29.8	12/25.5	5/10.6	1/2.1	0.997	0.910
Female	50/34.0	42/28.6	31/21.1	22/15.0	2/1.4		

### Comparison of coccygeal morphology for the same sex between the two groups

There were 156 males and 244 females in the two groups. The proportions of type I to type V patients who were male were 49.4%, 21.2%, 18.6%, 7.7% and 3.2%, respectively, and who were female were 39.3%, 24.2%, 18.0%, 16.4% and 2.0%, respectively. The difference in coccygeal morphology between the two groups was not statistically significant (*P* = 0.055; Table 6). Similarly, there was no significant difference in coccygeal morphology between the two groups. (*P* = 0.062; Table 7).

**Table 6:** Comparison of coccygeal morphology between male individuals in the AIS group and the normal group (cases/%).

	Type I	Type II	Type III	Type IV	Type V	χ2	*P*
Normal group	62/56.9	19/17.4	17/15.6	7/6.4	4/3.7	9.263	0.055
AIS group	15/31.9	14/29.8	12/25.5	5/10.6	1/2.1

**Table 7:** Comparison of coccygeal morphology between female individuals in the AIS group and the normal group (cases/%).

	Type I	Type II	Type III	Type IV	Type V	χ2	*P*
Normal group	46/47.4	17/17.5	13/13.4	18/18.6	3/3.1	0.849	0.065
AIS group	50/34.0	42/28.6	31/21.1	22/15.0	2/1.4		

### Correlations between coccygeal morphology and sex, scoliosis incidence, Cobb angle and scoliotic segment length

The correlations of coccygeal morphology with sex, scoliosis status, Cobb angle, and scoliotic segment were analyzed separately, and the results showed that coccygeal morphology was significantly correlated with scoliosis (*P* = 0.035); however, there were no significant correlations between coccygeal morphology and sex, Cobb angle, or scoliotic segment (Table 8).

**Table 8:** Correlation between coccygeal morphology and gender, scoliosis, Cobb angle and scoliotic segments.

	gender	scoliosis	Cobb angle	scoliotic segments
coefficient	0.098	0.105	0.046	0.026
P	0.050	0.035	0.528	0.724

## Discussion

Correlations between scoliosis and the cervical, thoracic, or lumbar spine have been reported previously^8–12^. However, as a component of the sagittal plane, there is currently no documented research on the difference in coccygeal morphology in AIS patients. The aim of this study was to observe and compare coccygeal morphology between non-scoliotic adolescents and AIS patients. Moreover, we analyzed the correlation of coccygeal morphology with scoliosis incidence, sex, Cobb angle, and the number of scoliotic segments.

### Coccygeal morphology in AIS patients

AIS is a prevalent three-dimensional spinal deformity, affecting 0.11 to 1.91% of individuals in China^20^. The incidence has gradually increased in recent years, and scoliosis has become a prevalent adolescent disease followed by obesity and myopia^21^. AIS is a kind of deformity associated with spinal changes in the coronal, horizontal and sagittal planes. Increasing numbers of researchers are interested in studying the sagittal spine in AIS patients. We compared coccygeal morphology between non-scoliotic adolescents and AIS patients in this research. The results showed that compared to the AIS group, the non-scoliotic group had a significantly larger proportion of type I patients; conversely, the proportions of type II and III patients were significantly larger in the AIS group. Type V is the least prevalent type for non-scoliotic adolescents and for scoliotic patients. Previous coccygeal morphology studies have shown that type V is the least prevalent among the different types^22–25^. These results confirm that coccygeal morphology is different in AIS patients, similar with the sagittal spine observed in AIS patients in previous studies^26,27^^.^ It has been suggested that the sacrococcygeal joint could be strained by constant pressure on the coccyx during an incorrect sitting posture^28^. Yuan et al.^29^ reported differences in hip pressure between the right and left sides in AIS patients. We speculate that the imbalanced hip pressure and incorrect sitting posture may be related to the differences in the coccygeal morphology of AIS patients.

In addition, several previous studies demonstrated that there was a correlation between scoliosis and paraspinal muscles^30–33^. An imbalance of paraspinal muscles in AIS patients results in the pelvic-spinal system being out of balance in the sagittal plane, thus causing spinal deformity progression^34,35^. A previous study also confirmed that unilateral contraction of some muscles, such as the gluteus maximus and coccygeus, could exert a pulling force on the coccyx laterally and anteriorly^36^. Although alterations in the pelvic muscles in AIS patients have not been fully elucidated at present, we believe that an imbalance in the paraspinal muscles in AIS patients may result in an imbalance of contraction of the pelvic muscles and subsequently change the coccygeal morphology. The difference in coccygeal morphology observed in AIS patients in our study may be related to the affected muscles around the coccyx, which were investigated in the above studies.

Several studies have confirmed a significant correlation between the scoliotic curve and decreased TK in AIS patients^26,37^. Moreover, the relationship between TK and LL in AIS patients has also been proven^38,39^. Legaye et al.^40^ suggested that sagittal pelvic parameters before surgery, such as the pelvic incidence (PI), could be used to estimate the ideal value of LL after surgery for spinal deformity. Furthermore, Mac-Thiong et al.^41^ suggested that the LL angle in normal adolescents was closely correlated with the sacral slope (SS) controlled by the PI. Two studies showed that the LL angle was more closely related to pelvic parameters in the sagittal plane. In addition, Kurnik et al.^42^ reported that an increased TK angle was associated with larger LL and cervical lordosis angles, which may lead to abnormal loads on the sacrum and coccyx. However, some studies have confirmed that the TK angle is smaller in AIS patients than in healthy individuals^37,43^, while the LL angle is greater in patients with lumbar scoliosis^17^. The above studies demonstrated that the TK and LL angles may directly or indirectly influence the growth of pelvic morphology, thus causing the structure and morphology of the sacrum and coccyx to change due to abnormal pressure. This was why we found significant differences in coccygeal morphology between the normal controls and AIS patients in this study.

Most non-scoliotic or scoliotic adolescents have type I despite the differences in coccygeal morphology; that is, type I was the most common type in adolescents with or without scoliosis. A study of coccygeal morphology in Arab adults revealed that type I was the most common type in the Arab population^22^. An analysis of a study by Woon JT et al. on a European population revealed that type I was also the most common type in the general population^23,24^. Additionally, a study by Kerimoglu U et al. revealed that type I was also the most common type of coccygeal morphology in the Turkish population^25^. These findings are consistent with the results of the present study, suggesting that type I is the most prevalent type among different populations and regions.

### Coccygeal morphology in patients with different types of scoliosis

According to previous research on patients with AIS, there is a typical correlation between scoliosis severity and the thoracic sagittal plane^44^. However, previous research on changes in the lumbar sagittal plane in AIS patients has yielded inconsistent results. Zhang et al. showed that compared to normal adolescents, AIS patients had significantly larger LL angles, which may be related to the type of scoliosis in AIS patients^27^. In contrast, some studies have shown that the LL angles in AIS patients are similar to those in healthy adolescents and that LL angles within the normal range is also similar among different types of scoliosis^45^. Patients with mild and moderate scoliosis were included in this study, and we found that type I was the most common type of mild scoliosis, followed by type III and type II. However, type II is most common in patients with moderate scoliosis. These results suggest that there are some differences in coccygeal morphology between patients with mild and moderate scoliosis, which are related to the severity of scoliosis. However, statistical analysis revealed no significant difference in coccygeal morphology between the two groups. We think that this difference may be related to the insufficient sample size of patients with moderate scoliosis in this study, and it is necessary to increase the sample size to further clarify these findings in the future.

In this study, we observed coccygeal morphology in different scoliotic segments and found that type I is the most common in different scoliotic segments. Several studies have concluded that TK is not significantly correlated with LL in patients with thoracic or lumbar segmental scoliosis^46^. In contrast, the study showed a correlation between TK and LL in patients with double thoracic scoliosis^47^. These different findings suggested that there was an association between scoliotic segments and spinal morphology in the sagittal plane, but this association may be based on specific pathways that have not yet been described. The results from our study also showed some distinctions in coccygeal morphology in the sagittal plane in different scoliotic segments. The difference in coccygeal morphology may be correlated with changes in the whole sagittal spine in AIS patients, and the relevant mechanism is worthy of further research.

### Correlation analysis of coccygeal morphology in AIS patients

Previous investigations have shown a clear correlation between sex and AIS incidence, with a significantly greater incidence of AIS in females than in males, especially in moderate and severe cases, with a ratio of 7:1 when the Cobb angle is 30° or greater^48,49^. Moreover, the correlation between AIS incidence and age suggested that older adolescents had a higher incidence of scoliosis^50^. Our study analyzed the correlation between coccygeal morphology and related factors, such as sex, scoliosis severity, and the number of scoliotic segments. Our findings indicate that there is no significant correlation between coccygeal morphology and these factors. There was no significant correlation between sex and AIS patients or normal adolescents. Our results are similar with those of several previous studies. Li et al. reported that overall pelvic parameters were not affected by sex, although the female pelvis was more susceptible to postural changes than the male pelvis^51^. Moreover, Janssen et al. demonstrated that pelvic parameters and coccygeal morphology were not significantly affected by sex^52^. However, by analyzing the results of their research on coccygeal morphology in adult Arabs, Marwan YA and his colleagues discovered that there was a correlation between coccygeal morphology and sex in normal individuals^22^. Another study by Zhu et al. showed that spinal parameters in the sagittal plane were notably different among individuals of different races^53^. Interestingly, these findings differ from our results, and we believe that this discrepancy could be attributed to variations in race. In addition, it has also been suggested that some spinal parameters in the sagittal plane tend to fluctuate with age^54^. These results are not in agreement with our research and may be related to the recruitment of subjects who were teenagers in our study. Although there were no significant correlations between coccygeal morphology and scoliosis severity or scoliotic segments in the present study, the proportions of type I to type V were different.

This was a retrospective study in which a crude statistical analysis of coccygeal morphology in AIS patients versus non-scoliotic adolescents was performed. The complexity of the coccyx classification system probably led to an insufficient sample size in some categories, which had an impact on the results to some degree. The coccygeal morphology in the coronal plane was not classified.

In summary, coccygeal morphology is significantly different between AIS and non-scoliotic adolescents. Compared to the non-scoliotic group, the AIS group had fewer patients with a type I coccyx and more patients with a type II or type III coccyx. In other words, the presence of a more pronounced coccygeal curve may be caused by an incorrect sitting position and an imbalance of contraction of the pelvic muscles in AIS patients. It should be further studied whether correcting the sitting position and muscular imbalances could change coccygeal morphology and subsequently affect the development of AIS.

## Materials and methods

### Study population

This was a retrospective observational study of the clinical data of AIS patients who underwent anteroposterior and lateral full-spine X-ray imaging at the 3rd Affiliated Hospital of Zhejiang Chinese Medical University between January 2018 and December 2023. To ensure high-quality data, we recruited adolescents aged 10 to 18 years who met the diagnostic criteria of the 2016 Guideline for the Management and Rehabilitation of Ischemia Scoliosis. During Growth recommended by the International Society on Scoliosis Orthopedic and Rehabilitation Treatment^18^. Patients with mild scoliosis with Cobb angles ranging from 10 to 25° and moderate scoliosis with Cobb angles ranging from 25 to 45° were recruited. Patients with any no idiopathic causes of spinal deformity were excluded. A total of 194 patients with a mean age of 14.14 ± 2.24 years (range, 10–18 years) and a mean Cobb angle of 20.21° ± 7.98° (range, 10.0°–42.4°) were recruited for the analysis. In addition, 206 patients without scoliosis and a mean age of 13.00 ± 2.20 years (range, 10–18 years) were enrolled in the control group. This study was approved by the Ethics Committee of the 3rd Affiliated Hospital of Zhejiang Chinese Medical University (ZCMU) (01/01/2018/No. ZSLL-KY-2017-045). Because secondary data were used, the need for informed consent was waived by the ethics committee of the 3rd Affiliated Hospital of ZCMU. The relevant guidelines and regulations performed all methods.

### Radiographic parameters

**Figure 1:**
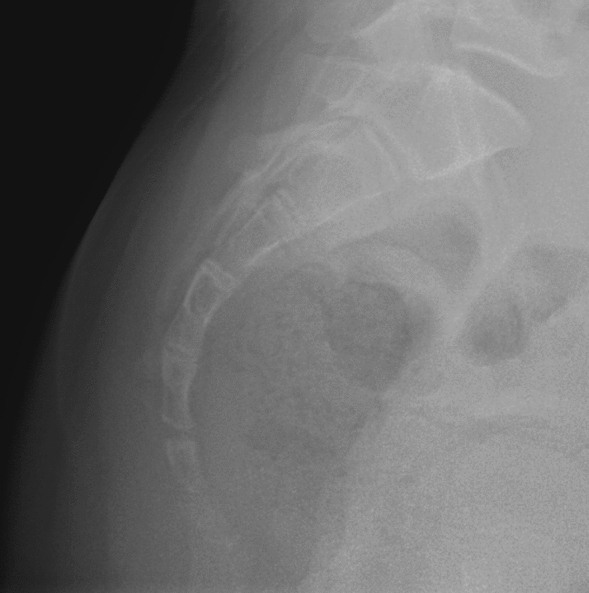
Type I based on Nathan’s modified Postacchini coccygeal radiological classification.

**Figure 2:**
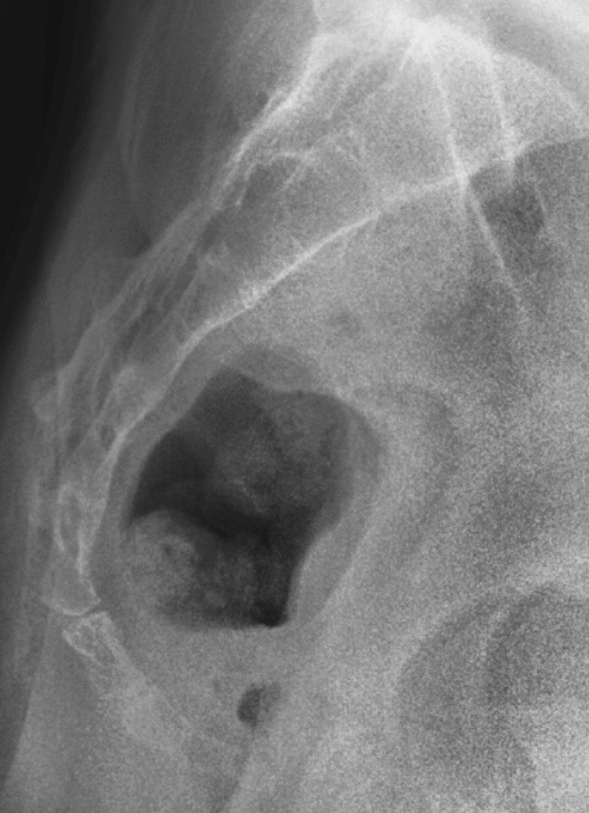
Type II based on Nathan’s modified Postacchini coccygeal radiological classification.

**Figure 3:**
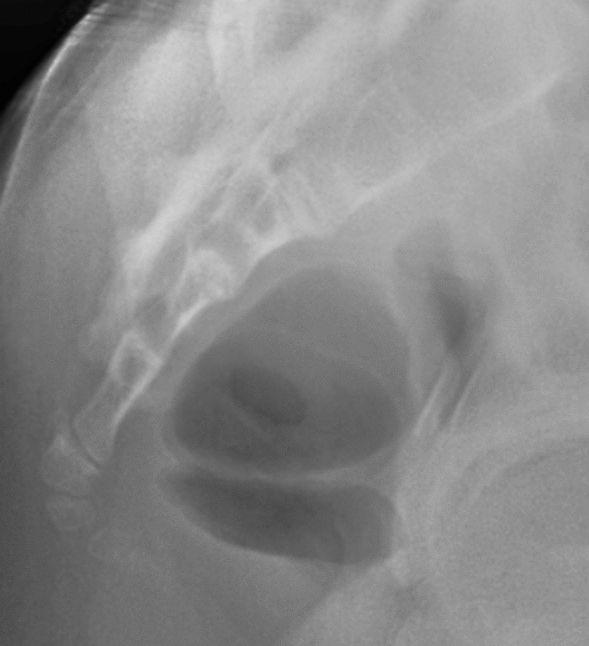
Type III based on Nathan’s modified Postacchini coccygeal radiological classification.

**Figure 4:**
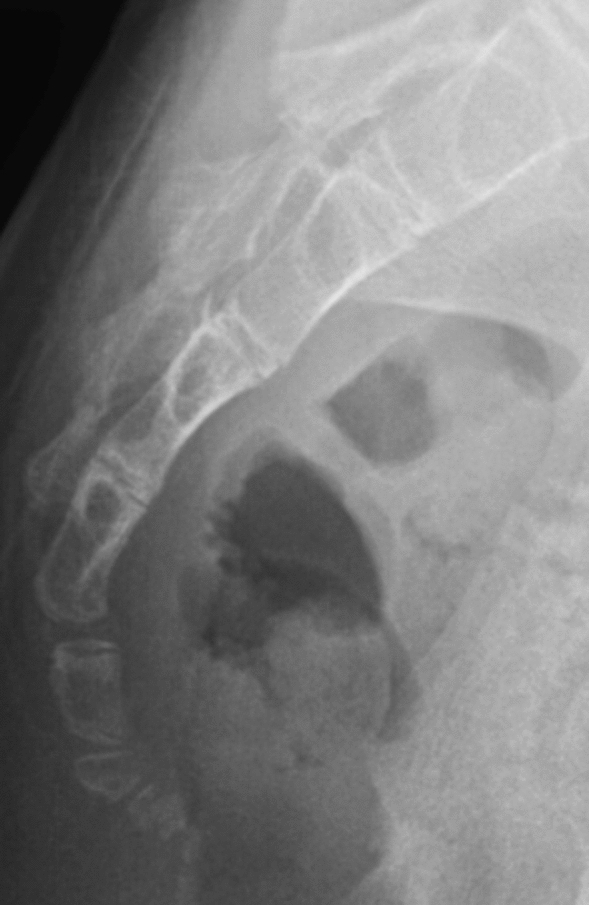
Type IV based on Nathan’s modified Postacchini coccygeal radiological classification.

**Figure 5:**
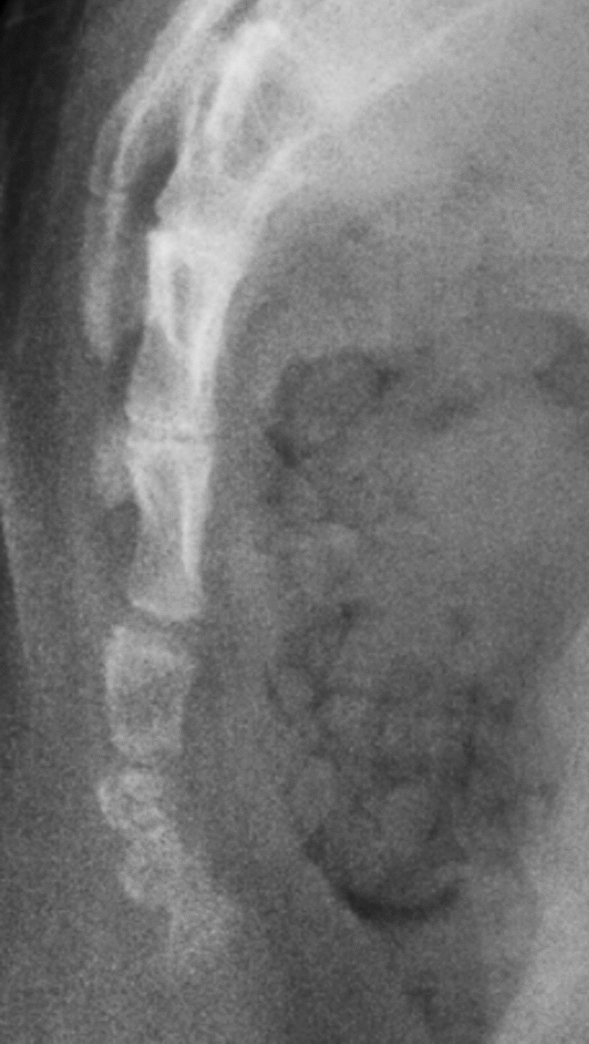
Type V based on Nathan’s modified Postacchini coccygeal radiological classification.

Anteroposterior and lateral X-rays of the spine were obtained in the standing position for all included subjects. The Cobb angle was measured on the anteroposterior X-rays and a Cobb angle ≥ 10° indicated scoliosis. Moreover, Nathan's modified Postacchini radiographic classification method was used to categorize the coccyx into five types in the sagittal plane^19^:(i)Type I: the coccygeal vertebra is slightly curved pointing downward(Fig. 1);(ii) Type II: the caudal vertebrae have a marked curve with the tip of the coccyx facing forward (Fig. 2);(iii) Type III: the 1st caudal vertebra is clearly angled to the 2nd caudal vertebra, or the 2nd caudal vertebra to the 3rd caudal vertebra (Fig. 3);(iv) Type IV: the caudal vertebra is displaced forward at the level of the sacrococcygeal joint or between the caudal 1/caudal 2 (Fig. 4);(v) Type V: the caudal vertebra is curved backward or has a small pin-like bone with the tip of the coccyx facing backward (Fig. 5).

### Statistical analysis

Statistical analyses were performed using IBM SPSS Statistics 25.0 software (SPSS, Inc., Chicago, IL, USA). The different types of coccygeal morphology were compared between groups using the chi-square test. Spearman's correlation coefficient was used to evaluate the correlation between coccygeal morphology and scoliosis type. A two-tailed *P* value < 0.05 was considered to indicate statistical significance.

## Data Availability

The datasets used and/or analyzed during the current study available from the corresponding author on reasonable request.
